# Cyclin-Dependent Kinase 1 Inhibition Potentiates the Proliferation of Tonsil-Derived Mesenchymal Stem Cells by Delaying Cellular Senescence

**DOI:** 10.1155/2022/4302992

**Published:** 2022-07-21

**Authors:** Da Hyeon Choi, Kyeong Eun Lee, Yoon Shin Park

**Affiliations:** Department of Biological Sciences and Biotechnology, School of Biological Sciences, College of Natural Sciences, Chungbuk National University, Cheongju 28644, Republic of Korea

## Abstract

Mesenchymal stem cells (MSCs) have been widely used in tissue regeneration and stem cell therapy and are currently being tested in numerous clinical trials. Senescence-related changes in MSC properties have attracted considerable attention. Senescent MSCs exhibit a compromised potential for proliferation; senescence acts as a stress response that prevents the proliferation of dysfunctional cells by inducing an irreversible cell cycle arrest. Here, we established a senescent MSC model using senescence-associated *β*-galactosidase, proliferation, and cell cycle assays. We further identified novel biomarker candidates for old, senescent tonsil-derived MSCs (TMSCs) using transcriptomics. A plot of the cellular senescence pathway showed cyclin-dependent kinase 1 (CDK1; +8-fold) and CDK2 (+2-fold), and transforming growth factor beta 2 (TGFB2; +2-fold) showed significantly higher expression in old TMSCs than in young TMSCs. The CDK family was shown to be related to cell cycle and proliferation, as confirmed by quantitative RT-PCR. As replicative senescence of TMSCs, the gene and protein expression of CDK1 was significantly increased, which was further validated by inhibiting CDK1 using an inhibitor and siRNA. Taken together, we suggest that the CDK1 can be used as a selective senescence biomarker of MSCs and broaden the research criteria for senescent mechanisms.

## 1. Background

Cellular senescence-related changes in mesenchymal stem cell (MSC) properties have attracted considerable attention in the tissue engineering and regenerative medicine fields [1, 2]. Senescent MSCs exhibit a compromised potential for proliferation, homing effective migration, tissue differentiation, angiogenesis, and regeneration [3–6]. However, therapies utilizing MSCs often require ex vivo expansion to generate large quantities of cells required for patients [7–9]. Despite their therapeutic potential and safety, MSCs must be serially passaged for an extended period to obtain a sufficient number of cells for *in vivo* or clinical application [10].

Long-term replicative subculture of MSCs induces continuous changes in cell properties, including decreased proliferation rate, self-renewal, and regenerative potential, resulting in cell cycle arrest and changes in morphology such as cell widening and flattened shape [8, 11, 12]. Cellular senescence occurs in response to DNA damage, oncogene mutations, oxidative stress, damage-associated molecular pattern molecules from diseased tissue, and various other cellular insults [13–15]. In particular, senescence has been well characterized as a stress response that prevents the proliferation of dysfunctional cells by inducing irreversible cell cycle arrest [16, 17].

During long-term replicative cell culture, MSCs may undergo molecular changes that result in the acquisition of senescent phenotypes independent of MSC isolation and culture conditions [18]. To achieve a successful regenerative outcome, an increasing number of studies have recently highlighted the roles of cell cycle-related genes or proteins in therapeutic MSCs because they act as key contributors during proliferation largely by regulating cell cycle responses [16]. Additionally, optimized strategies need to be developed to elucidate the cell cycle regulating factors in senescent MSCs for better outcomes in stem cell therapy [19].

Recently, tonsil-derived MSCs (TMSCs) obtained from human tonsil tissues, generally isolated from waste tissues after a tonsillectomy, have been applied as MSC sources for clinical applications owing to the noninvasive nature of tissue collection without unnecessary surgeries and high proliferation rate [20]. Regardless of their source, replicative subculture also leads to a decrease in TMSC functions by leading them to a state of replicative senescence (i.e., *in vitro* culture aging, also known as the Hayflick limit) [21].

Furthermore, MSCs undergo senescence after a certain number of cell expansion passages *in vitro* and finally stop the proliferation [22]. Senescent MSCs often exhibit persistent activation of the DNA damage response with concomitant increases in cyclin-dependent kinase (CDK) inhibitors [23]. CDKs regulate various proteins necessary for cell cycle progression and prevent advancement when bound to their cyclin partners [24]. The CDK inhibitors p16INK4a (encoded by CDKN2A) and p21 (encoded by CDKN1A) have been highly implicated in senescence, along with the p53 signaling pathway [25, 26]. Specifically, p16 inhibits G1 to S phase transition via inhibition of CDK4 and CDK6. The p21 acts similarly via inhibition of CDK2, among other CDKs [27]. Therefore, it is necessary to understand the association of CDK with the cell cycle to promote the proliferation of senescent MSCs.

In this study, we established a new *in vitro* senescent stem cell model using various experiments, including the senescence-associated *β*-galactosidase (SA-*β*-gal) assay, proliferation assay, and cell cycle assay. Additionally, we investigated differential gene expression patterns between young and old TMSC groups, distinguished by long-term replicative cultivation. Using transcriptomic approaches, we identified novel biomarker candidates for detecting old, senescent TMSCs, thereby elucidating the role of identified biomarkers in the functional recovery of old TMSCs.

## 2. Materials and Methods

### 2.1. Isolation and Culture of TMSCs

TMSCs were isolated from excised palatine tonsillar tissues obtained from four patients following tonsillectomy, as described previously [28]. Tonsillar tissues used in this study were acquired from four donors (2 girls and 2 boys) who had undergone a tonsillectomy due to tonsil hyperplasia without tonsillitis. In addition, the donors consisted of patients under 10 years of age (Supplementary Table 1). Before surgery, all subjects provided their informed written consent for the use of their tissue specimens for research purposes and TMSC isolation according to the guidelines of the Chungbuk National University Medical Center (IRB No. CBNU-202106-BRHR-0073). Tonsillar tissues used in this experiment were collected from patients who underwent tonsillectomy due to tonsil hyperplasia.

The isolated tonsils were mechanically digested by cutting, and the tissues were then chemically digested using collagenase type I (Thermo Fisher Scientific, Waltham, MA, USA) and DNase (Sigma-Aldrich, St. Louis, MO, USA) at 37°C for 30 min. The obtained cell suspension was filtered through a wire mesh, and mononuclear cells were isolated using Ficoll-Paque (GE Healthcare, Piscataway, NJ, USA) density gradient centrifugation. The resulting mononuclear cells were cultured in DMEM-HG (Welgene Inc., Gyeongsan, Korea) supplemented with 10% fetal bovine serum (FBS: certified, US origin, Gibco, Grand Island, NY, USA), 1% antibiotics/antimycotics (A/A), and penicillin/streptomycin (P/S) (Gibco) at 37°C in a humidified 5% CO_2_ incubator.

Cells were allowed to adhere to culture plates for 24 h, and adherent mononuclear cells were used as TMSCs. The cells were cultured in DMEM supplemented with 10% FBS, and the medium was changed every two days. When the TMSCs reached 80–90% confluence, they were treated with 0.25% trypsin-EDTA (Gibco) for 3 min. The detached cells were washed twice with PBS and collected by centrifugation at 3,000 rpm for 5 min (Eppendorf, Hamburg, Germany).

### 2.2. Experimental Groups of TMSCs

After reaching 80% confluence, TMSCs were subcultured onto individual dishes and replicative subcultured to passages 15 to 20 to induce cellular replicative senescence. The maximum number of passages was determined by the proliferative capacity. The TMSCs used in this study were between passages 5 and 20. The growth medium was changed every three days. The *in vitro*-cultured TMSCs were divided into two groups as follows: young (passages 5–8) and old (passages 15–20) TMSCs.

### 2.3. Senescence-Associated-*β*-Gal Assay

Morphological changes associated with experimental treatments, including increased cell size, altered overall morphology, and decreased proliferative capacity, were assessed with an inverted microscope (Olympus). Senescent TMSCs were detected by SA-*β*-gal staining using a commercial staining kit (Cell Signaling Technology, Boston, MA, USA) according to the manufacturer's instructions. Briefly, TMSCs were fixed with 4% paraformaldehyde (Biosesang, Seongnam, Korea) for 15 min at 37°C, and the fixed cells were incubated with *β*-gal staining solution at 37°C overnight in a dry incubator without CO_2_ supply. Old cells were identified by their *β*-gal blue-stained cells under a standard light microscope. Stained cells were expressed as a percentage of total TMSCs.

### 2.4. Cell Cycle and Proliferation Analysis

The cell cycle stage was examined by measuring the DNA content of nuclei labeled with propidium iodide (PI, Abcam). For the cell cycle analysis, either young or old TMSCs (1 × 10^5^ cells) were harvested by trypsinization and gently pelleted by centrifugation at 300 × *g* for 5 min. The pellets were washed and resuspended in 200 *μ*l DPBS. The resuspended cells were transferred dropwise into 70% ethanol (800 *μ*l) and fixed for 1 week. The fixed cells were collected, washed, and resuspended in PI staining solution (50 mg/ml) containing RNase A (100 mg/ml) and incubated in the dark for 30 min at room temperature. A FACScan (FACSCalibur-S System; BD Biosciences) was used to analyze the cell cycle, and the FlowJo software was used for data analysis.

### 2.5. Cell Proliferation Assay

For the proliferation assay, TMSCs were seeded at a density of 1.5 × 10^5^ cells/well in a 6-well plate, and plates were transferred to the automated real-time live cell imaging system (IncuCyte, Essen BioScience, MI, USA). CDK1-inhibited/transfected old TMSCs have been applied to the IncuCyte system. The changes of cell confluence with treatment were monitored and measured by the IncuCyte software, based on the five different spot, and the changes of cell populations were counted every 2 h for 37 h.

### 2.6. Fluorescence-Activated Cell Sorting (FACS) Analysis

The TMSCs were phenotypically characterized by flow cytometry. The TMSCs (1.0 × 10^4^ cells) from the two experimental groups were incubated with fluorescein isothiocyanate- (FITC-) or phycoerythrin- (PE-) conjugated monoclonal antibodies against isotype-PE, isotype-FITC, CD14, CD34, CD45, CD73, CD90, and CD105 (BD Biosciences, San Jose, CA, USA) for 30 min at 4°C. Cell populations were analyzed using a FACScan instrument (FACSCalibur-S). Nontreatment TMSCs, isotype-PE, and isotype-FITC Ig control for each wavelength were used as a control. Data were analyzed using FlowJo (BD Biosciences). Results were displayed as the percentage of labeled cells for each monoclonal antibody.

### 2.7. RNA Quality Assessment

TMSCs isolated from four donors (two boys and two girls, under 10 years old) with different passage numbers were used for the assessment of RNA quality. RNA used for microarray analysis was obtained from young and old passages from each of the two donors (*n* = 2 per group). RNA was isolated from TMSCs using the TRIzol reagent (Thermo Fisher Scientific). RNA purity (260/280 ratio) and RNA integrity number (RIN) were evaluated using an ND-1000 spectrophotometer (NanoDrop, Wilmington, Delaware, USA) and Agilent 2100 Bioanalyzer (Agilent Technologies, Palo Alto, California, USA), respectively. RNA samples with values higher than 1.7 for RNA purity and 7.0 for RIN were used for the microarray analyses.

### 2.8. Affymetrix Whole Transcriptomic Arrays

The Affymetrix whole transcript expression array process was executed according to the manufacturer's protocol (GeneChip WT PLUS Reagent Kit, Thermo Fisher Scientific). cDNA was synthesized using the GeneChip WT Amplification Kit, as described by the manufacturer. The sense cDNA was then fragmented and biotin-labeled with TdT (terminal deoxynucleotidyl transferase) using the GeneChip WT terminal labeling kit. Approximately 5.5 *μ*g of labeled cDNA was hybridized to the Affymetrix GeneChip Human Clariom D array at 45°C for 16 h. Hybridized arrays were washed and stained on a GeneChip fluidics station 450 and scanned on a GCS3000 scanner (Thermo Fisher Scientific). Signal values were computed using Affymetrix GeneChip Command console software 5.0 (Thermo Fisher Scientific). All transcriptomic data are listed in GEO accession number GES149588.

### 2.9. Preparation and Statistical Analysis of the GO Term and KEGG Pathway

Data were summarized and normalized using the robust multiaverage (RMA) method implemented in Affymetrix Power Tools. The results of gene-level RMA analyses were exported to analyze differentially expressed genes (DEGs). The false discovery rate was controlled by adjusting the *p* value using the Benjamini-Hochberg algorithm. For a given DEG set, hierarchical cluster analysis was performed using the complete linkage and Euclidean distance as a measure of similarity. Gene enrichment and functional annotation analyses of significant probe lists were performed using the GO and KEGG databases. The statistical significance of the expression data was determined using a local-pooled-error test and measurements of fold change (fc), with the null hypothesis that no difference exists between groups. All data analyses and visualization of DEGs were conducted using R 3.0.2.

### 2.10. CDK1 Knockdown Experiments Using Inhibitor and siRNA Transfection

CDK1 protein expression in old TMSCs was knocked down using an inhibitor and siRNA. Two days before treatment, the old TMSCs were grown in 6-well plates containing DMEM supplemented with 10% FBS. The CDK1/2 inhibitor III (Enzo Life Sciences, Inc., NY, USA) was used at the 10 *μ*M concentration in DMSO, which is sufficient to induce robust checkpoint arrest in cells at the cell cycle stage. CDK1 siRNA and scrambled siRNA were purchased from Santa Cruz Biotechnology (Santa Cruz). The scrambled control siRNA or CDK1 siRNA was transfected into the old TMSCs using Lipofectamine 2000 and LipoPLUS (Thermo Fisher). After the transfection, the cell lysates were collected and analyzed using quantitative RT-PCR analysis.

### 2.11. Quantitative RT-PCR

TMSCs were plated onto 100 mm^2^ culture dishes and incubated at 37°C under 5% CO_2_ till they reached 80–90% confluence. Total RNA from TMSCs was extracted using the TRIzol reagent according to the manufacturer's instructions (Life Technologies, Darmstadt, Germany). Isolated RNA was treated with DNase I (Thermo Scientific, Schwerte, Germany) to remove possible genomic DNA contamination and used for cDNA synthesis using Superscript III Transcriptase (Life Technologies) and random hexamer primers (Thermo Scientific) at 50°C for 1 h. Each sample was run in triplicate. Quantitative RT-PCR was conducted using POWER SYBR Green qPCR Master Mix (Life Technologies) and 0.2 *μ*M primer. The primer sequences used were as follows: (1) TRF1 (200 bp): forward, 5′-CCA CAT GAT GGA GAA AAT TAA GAG TTA T-3′, and reverse, 5′-TGC CGC TGC CTT CAT TAG A-3′; (2) hTERT (200 bp): forward, 5′-CGG AAG AGT GTC TGG AGC AA-3′, and reverse, 5′-GGA TGA AGC GGA GTC TGG A-3′; (3) CDK1 (200 bp): forward, 5′-TGG AGA AGG TAC CTA TGG AGT TG-3′, and reverse, 5′-AGG AAC CCC TTC CTC TTC AC-3′; (4) CDK2 (190 bp): forward, 5′-AAA GCC AGA AAC AAG TTG ACG-3′, and reverse, 5′-GAG ATC TCT CGG ATG GCA GT-3′; (5) TGFB2 (200 bp): forward, 5′-CTG ACG GCC ACG AAC TTC C-3′, and reverse, 5′-GCA CTG ACA TTT GTC CCT TGA-3′; (6) CCNA2 (204 bp): forward, 5′-ATG TCA GCG ATA TCC ACA CG-3′, and reverse, 5′-GCT CCA TCC TCA GAA CTT GC-3′; (7) CCNB1 (200 bp): forward, 5′-CTG TTG GTT TCT GCT GGG TGT-3′, and reverse, 5′-CGC CTG CCA TGT TGA TCT TCG-3′; (8) CCNE2 (200 bp): forward, 5′-ACC TCA CTC TTC ATT GCC TCC-3′, and reverse, 5′-TCA CAA ACG GAA CCA TCC AC-3′; and (9) *β*-actin (200 bp), which was used as a control: forward, 5′-CGA GCG CGG CTA CAG CTT-3′, and reverse, 5′-CCT TAA TGT CAC GCA CGA TTT-3′. The PCR was conducted using a StepOne Plus Real-Time PCR System (Applied Biosystems, Foster City, CA) under the following cycling conditions: initial denaturation at 95°C for 5 min, 40 denaturation cycles of 30 s at 95°C, and annealing for 40 s at 60°C and 30 s at 72°C, followed by fluorescence measurements.

### 2.12. Western Blot Analysis

TMSCs were lysed with a RIPA buffer containing protease inhibitor mixture (Roche Applied Science). Equal amounts of protein were loaded and separated by sodium dodecyl sulfate-polyacrylamide gel electrophoresis. The separated proteins were transferred onto nitrocellulose membranes with 0.2 *μ*m pore size (GE Healthcare). The membranes were then blocked in TBST with 5% skim milk, and the blots were incubated with appropriate primary antibodies followed by the corresponding secondary antibodies tagged with HRP, which were then visualized using enhanced chemiluminescence reagents. Primary antibodies against the following proteins were used in this study: CDK1, CDK2, cyclin B1, cyclin E2 (Cell Signaling Technology), and GAPDH (AB Frontier). All protein band images from western blot analyses were quantified densitometrically using ImageJ software (National Institutes of Health, Bethesda, MD, USA).

### 2.13. Statistical Analysis

All data are presented as the mean ± standard deviation (SD). All data graphs were generated using the GraphPad Prism 7 software (GraphPad Software Inc., La Jolla, CA, USA). The significance of differences between the two experimental groups (young and old TMSCs) was analyzed using Student's *t*-test. Statistical significance was set at *p* value < 0.05 (^∗^*p* < 0.05, ^∗∗^*p* < 0.01, or ^∗∗∗^*p* < 0.001).

## 3. Results

### 3.1. Confirmation of Replicative Senescent TMSCs

**Figure 1:**
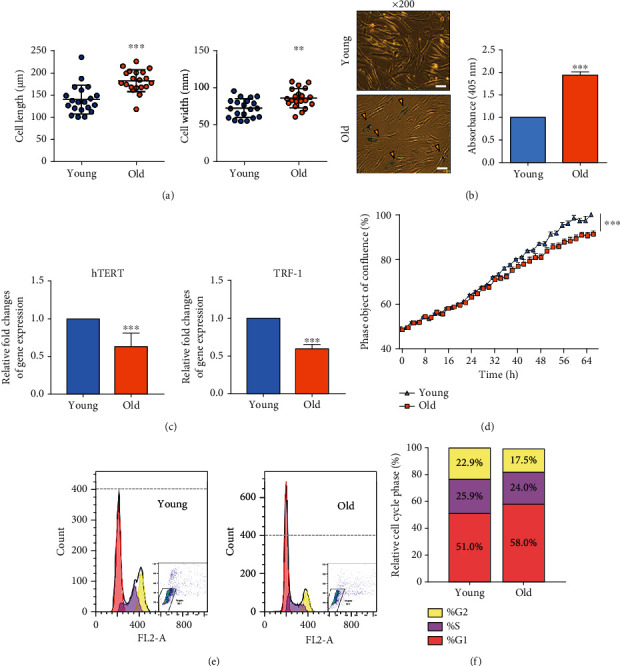
Changes in senescent markers with serial passaging of TMSCs. (a) Morphological changes of young and old TMSC groups were determined by measuring cell length and width. Young TMSCs exhibited a polygonal morphology, whereas old TMSCs formed dispersed shapes. (b) Senescent cells were identified as blue-stained cells under an optical microscope. Magnification: ×200; scale bar, 50 *μ*m. The orange arrowhead indicates stained cells. Senescent cells were identified as blue-stained cells and were calculated by the average absorbance intensity of SA-*β*-gal staining (405 nm) from 5 randomly selected fields between young and old TMSCs. (c) Gene expression of hTERT and TRF-1 which are telomere length markers in TMSCs. (d) Cell proliferation profile of young and old TMSCs during 64 h. The blue triangle line indicates young TMSCs, and the orange rectangle line indicates old TMSCs. (e, f) The TMSCs from each experimental group were stained with PI staining solution and analyzed their cell cycle stage. The *p* values were considered statistically significant at ^∗∗^*p* < 0.01 and ^∗∗∗^*p* < 0.001.

Old TMSCs were confirmed using the SA-*β*-gal assay, as well as by assessing morphological changes that became enlarged, dispersed, and flattened (Figures 1(a) and 1(b)). Compared to the old TMSCs, young TMSCs exhibited a polygonal morphology. Old TMSCs presented significantly longer lengths (140.48 ± 31.33 vs. 182.26 ± 23.93 *μ*m, *p* < 0.001) and wider widths (72.94 ± 12.08 vs. 86.13 ± 12.34 *μ*m, *p* < 0.01) than young TMSCs (Figure 1(a)). SA-*β*-gal staining revealed a higher intensity of X-gal staining among the culture-aged old TMSCs compared to that in the young group (Figure 1(b)). A quantitative analysis of the old TMSCs revealed an approximately 3.0 ± 0.2-fold increase compared to young cells (*p* < 0.001). Furthermore, quantitative RT-PCR analyses revealed that the old TMSCs presented significantly lower expression of senescence-related genes compared to the young cells; for instance, the expression of human telomerase reverse transcriptase (hTERT) and telomere regulation factor- (TRF-) 1 gene in the old TMSCs was 0.6 ± 0.3- and 0.6 ± 0.1-fold lower than those in the young TMSCs (Figure 1(c)), respectively. The real-time proliferation rate of old cells was significantly lower than that of young cells (*p* < 0.001, Figure 1(d)). As shown in Figures 1(e) and 1(f), we also found that the replicative senescence affected the cell cycle. The represented G1 phase increased from 51.8% to 58.0%, while the S and G2 phases decreased from 25.9% to 24% and from 22.9% to 17.5%, respectively.

### 3.2. Changes of Surface Marker Expressions with Senescence

**Figure 2:**
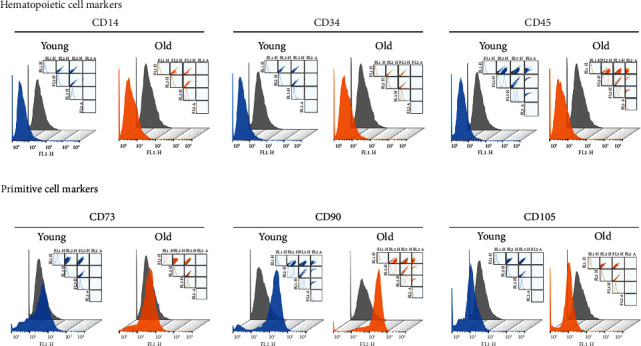
Changes in the expression of stem cell surface markers in TMSCs with serial passaging. Surface markers of TMSCs were characterized using FACS analysis. Both hematopoietic (CD14, CD34, and CD45) and primitive (CD73, CD90, and CD105) cell surface markers were examined. Young and old TMSCs were marked as blue and orange peaks, respectively.

The phenotypic characteristics of old TMSCs were assessed using representative stem cell surface markers, including hematopoietic cell markers (CD14, CD34, and CD45) and primitive cell markers (CD73, CD90, and CD105) (Figure 2). Using fluorescence-activated cell sorting (FACS), we found no significant changes in the expression of either hematopoietic or primitive surface protein markers between young and old TMSCs. Young (orange peak) and old TMSCs (blue peak) resulted in completely overlapped peaks, indicating that senescent old cells cannot be distinguished from young cells by using these surface markers.

### 3.3. Transcriptomic Profiles of Cellular Senescence in Young and Old TMSCs

**Figure 3:**
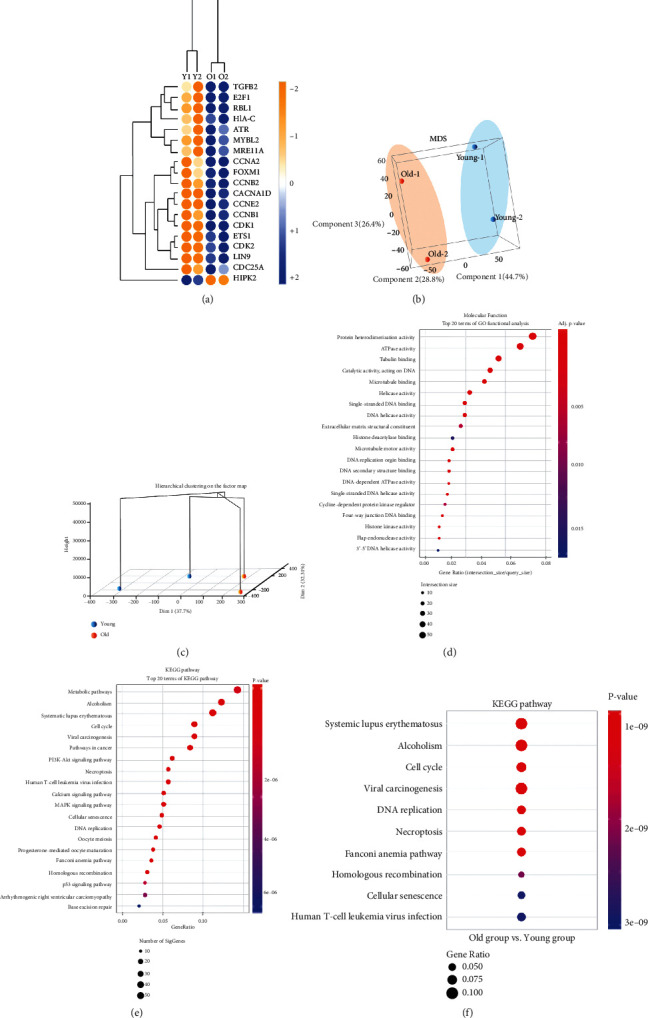
Transcriptomic profiles of young and old TMSCs. Whole-genome sequence data from young and old TMSCs are presented. (a) A cell cycle-related partial heatmap of hierarchical clustering analysis results indicates differentially expressing genes (rows) between the young and old TMSCs. Blue and orange circles indicate up- and downregulated genes, respectively, in old TMSCs compared with young TMSCs. For all comparisons, changes in gene expression are depicted as a heatmap. (b) 3D MDS plots for microarray gene expression data compared to young and old TMSCs were presented. (c) 3D dendrogram depicts the results of hierarchical clustering analysis of the interclass correlation between young and old TMSCs, confirming the classification of an interclass between the two groups. (d) The molecular function distribution of GO terms for DEGs between young and old TMSCs was annotated according to the ontology categories: molecular function (MF). The *x*- and *y*-axes indicate the number of DEGs and GO term gene classification, respectively (^∗∗∗^*p* < 0.001). (e) All KEGG pathways were first classified into 10 categories. (f) These categories were the top 20 terms of the KEGG pathway according to the level of significance.

We performed a transcriptomic analysis of young and old TMSCs. Senescence-related transcriptomic differences were presented as a log2 fold change heatmap distribution (|log2 (fold − change)| >2) in Figure 3(a). A complete list of heatmap genes is provided in Table 1. The quality of the DNA-seq data was confirmed by a repeatability check using Pearson's correlation coefficient analysis (*p* < 0.05). Based on multidimensional scaling (MDS) scores, the old group was distinguishable from the young group (Figure 3(b)). Unsupervised hierarchical clustering showed a clear distinction between the young and old TMSCs (Figure 3(c)).

### 3.4. Molecular Functional Gene Ontology of Highly Regulated Genes and KEGG Pathway Analysis in Old TMSCs

We categorized differentially expressed genes in old TMSCs according to the molecular function-related gene ontology (GO) classification (MF; Figure 3(d)). The top 20 enriched molecular function (MF) terms were protein heterodimerization activity, ATPase activity, tubulin binding, catalytic activity acting on DNA, microtubule binding, helicase activity, single-stranded DNA binding, DNA helicase activity, extracellular matrix structural constituent, histone deacetylase binding, microtubule motor activity, DNA replication origin binding, DNA secondary structure binding, DNA-dependent ATPase activity, single-stranded DNA helicase activity, cyclin-dependent protein kinase regulator, four-way junction DNA binding, histone kinase activity, flap endonuclease activity, and 3′-5′ DNA helicase activity.

To evaluate signaling pathways associated with enriched genes in replicative senescence of old TMSCs, we performed a KEGG pathway analysis (Figure 3(e)). The top 20 terms of KEGG pathway were listed as metabolic pathways, alcoholism, systemic lupus erythematosus, cell cycle, viral carcinogenesis, pathways in cancer, PI3K-Akt signaling pathway, necroptosis, human T cell leukemia virus infection, calcium signaling pathway, MAPK signaling pathway, cellular senescence, DNA replication, oocyte meiosis, progesterone-mediated oocyte maturation, Fanconi anemia pathway, homologous recombination, p53 signaling pathway, arrhythmogenic right ventricular cardiomyopathy, and base excision repair (Figure 3(f)).

To place these results in the context of cell cycle changes in old TMSCs and their proliferation rate, we focused on changes in the cell cycle, namely, cell cycle, cellular senescence, and p53 signaling pathway. A total of 35 cell cycle-related genes (*CDC20, CDKN2C*, *CDC7*, *TGFB2*, *MAD2L2*, *ORC1*, *BUB1*, *MCM6*, *CDC25A*, *ATR*, *MAD2L2*, *CCNA2*, *CCNB1*, *PTTG1*, *CDC25C*, *TTK*, *MCM3*, *DBF4*, *MCM7*, *MCM4*, *CCN2*, *RAD21*, *CDK1*, *ESPL1*, *CDK2*, *CCNB2*, *BUB1B*, *PLK1*, *ORC6*, *CDC6*, *PCNA*, *E2F1*, *RBL1*, *CDC45*, and *MCM5*), 19 cellular senescence genes (*TGFB2*, *LIN9*, *CACNA1D*, *CDC25A*, *ATR*, *CCNA2*, *CCNB1*, *HLA-C*, *HIPK2*, *CCNE2*, *CDK1*, *MRE11A*, *ETS1*, *CDK2*, *FOXM1*, *CCNB2*, *MYBL2*, *E2F1*, and *RBL1*), and 11 p53 signaling pathway-related genes (*RRM2*, *STEAP3*, *CASP8*, *ATR*, *CCNB1*, *CCNE2*, *CDK1*, *SESN3*, *CDK2*, *CCNB2*, and *GTSE1*) were identified (Supplementary Table 2).

Among the three pathways, we focused on the cellular senescence KEGG pathway (Supplementary Figure 1). The identified genes related to cellular senescence and proliferation from the in silico analyses were *CDK1*, *CDK2*, and transforming growth factor beta 2 (*TGFB2*), all of which indicate representative cellular senescence-specific pathways. A plot of cellular senescence KEGG pathway showed that *CDK1* (+8-fold change, fc), *CDK2* (+2-fc), and *TGFB2* (+2-fc) were significantly higher in old TMSCs than in young TMSCs. The gene that presented the most significant changes in expression with respect to senescence (>8-fc) was *CDK1*, shown to be related to the cell cycle and cell proliferation (Supplementary Table 2).

### 3.5. Expression of the Cellular Senescence and Cell Cycle-Related Genes

**Figure 4:**
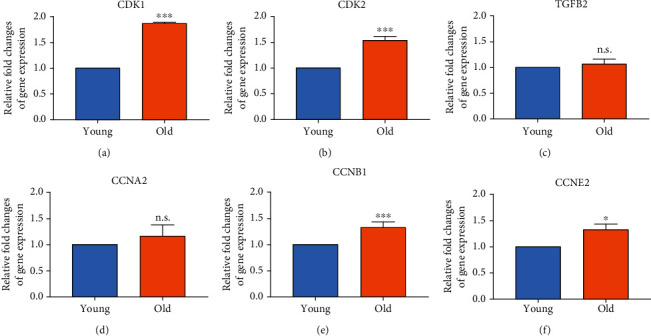
Changes of CDK family gene expression with replicative senescence in TMSCs. Changes in the expression of cellular senescence and cell cycle gene induced by replicative subculture were confirmed by quantitative RT-PCR (a) *CDK1*, (b) *CDK2* (c) *TGFB2*, (d) *CCNA2*, (e) *CCNB1*, and (f) *CCNE2*. The bar graph represents relative fold changes in gene expression, determined by cT value of each sample normalized to that of respective *β-actin*. The *p* values were considered statistically significant (^∗∗∗^*p* < 0.001, ^∗^*p* < 0.05; n.s.: not significant).

Changes of gene expression associated with senescence were determined using transcriptomics and confirmed by quantitative RT-PCR analyses for *CDK1*, *CDK2*, *TGFB2*, *CCNA2*, *CCNB1*, and *CCNE2* genes involved in cellular senescence, cell cycle, and p53 signaling pathways, respectively (Figure 4). Due to the senescence caused by serial passaging, the *CDK1* and *CDK2* gene expressions significantly increased to 1.86 ± 0.02-fold (*p* < 0.001, Figure 4(a)) and 1.53 ± 0.07-fold (*p* < 0.001, Figure 4(b)) compared to young cells, respectively, whereas there was no significant change in *TGFB2* and *CCNA2* gene expression (Figures 4(c) and 4(d)). In addition, compared to young TMSCs, *CCNB1* and *CCNE2* gene expressions increased to 1.45 ± 0.18-fold (*p* < 0.01, Figure 4(e)) and 1.32 ± 0.10-fold (*p* < 0.05, Figure 4(f)), respectively.

### 3.6. Changes of Cell Cycle-Specific Protein Expression and CDK1 Activity in TMSCs

**Figure 5:**
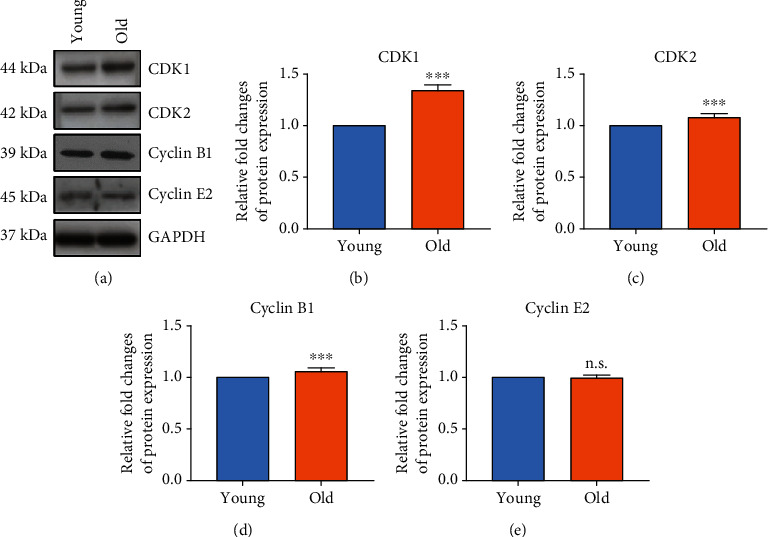
Changes of CDK family protein expression with replicative senescence in TMSCs. (a) Western blot analysis of cell cycle markers which are (b) CDK1, (c) CDK2, (d) cyclin B1, and (e) cyclin E2. The bar graph represents relative fold changes in gene expression, normalized by respective GAPDH. The *p* values were considered statistically significant (^∗∗∗^*p* < 0.001, ^∗^*p* < 0.05; n.s.: not significant).

As the replicative senescence of TMSCs progressed, the changes in cell cycle-specific marker protein were examined by western blot analysis of CDK1, CDK2, cyclin B1, and cyclin E2 (Figure 5). During replicative senescence, the CDK1 and CDK2 protein expression was significantly increased 1.3 ± 0.2-fold and 1.2 ± 0.1-fold compared to young TMSCs, respectively. There was no significant change in cyclin B1 and cyclin E2 expression between the two groups in protein expression. Between the CDK1 and CDK2 proteins, CDK1 was selected as a strong senescence marker candidate for further analysis because it presented higher level of significance.

### 3.7. Efficacy of CDK1 Protein Expression Activity in Inhibited Condition

**Figure 6:**
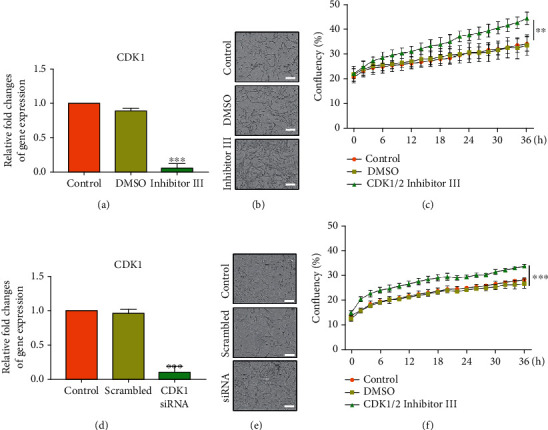
Confirmation of CDK1 function in TMSCs. The relative fold change bar graph of CDK1 gene expression with (a) CDK1/2 inhibitor III and (d) CDK1 siRNA. (b, e) Comparison of cell confluency for each group. Cell proliferation profile of (c) old TMSCs *vs.* CDK1-inhibited TMSCs and (f) old TMSCs *vs.* old TMSCs transfected with CDK1 siRNA. The *p* values were considered statistically significant (^∗∗∗^*p* < 0.001); scale bar, 200 *μ*m.

To investigate the efficacy of CDK1 to cell proliferation, the real-time proliferation rates of the old TMSCs and CDK1-inhibited/transfected TMSCs were calculated. As shown in (Figures 6(a)–6(c)), it was confirmed that cell proliferation was significantly increased compared to the old TMSC control group in the CDK1/2-inhibited group (*p* < 0.01). In addition, the group treated with DMSO used as a solvent showed a similar growth rate trend as the old TMSC control group. It was indicated that CDK1/2 affects the proliferation rate in old TMSC. Additionally, we performed the transfection of CDK1 siRNA to investigate whether CDK1 has its own efficacy (Figures 6(d)–6(f)). The knockdown of CDK1 in old TMSCs significantly influenced cell proliferation compared to old TMSCs in doubling time (*p* < 0.001).

## 4. Discussion

As evidence continues to emerge regarding the importance of improving MSC proliferation and clinical therapy, MSC therapy is becoming an increasingly attractive alternative or additive for the treatment of various diseases [29]. Human tissue-derived MSCs, including adipose-derived MSCs and bone marrow-derived MSCs, are ideal candidates for diverse regenerative medicine and tissue engineering strategies [30]. TMSCs have also been used for regeneration in various tissues, including bone [31], skeletal muscle [32], nerve [33], and parathyroid tissue [34]. To use MSCs for various clinical applications, replicative senescence is inevitable. However, many therapies involving MSCs often require ex vivo expansion approaches to generate the large number of cells required for the patients and to overcome the limitations of cell senescence [7].

Replicative senescence is defined as an irreversibly restricted proliferation due to telomere shortening in MSCs after a stereotypical number of cell divisions [35]. MSCs from elderly individuals recapitulate most parameters seen in senescent MSCs, including a flat, enlarged morphology, a large number of cells stained positive for SA-*β*-gal, and a lower proliferation rate. These characteristics have fueled the perception that replicative senescence *in vitro* may serve as a candidate model to unravel the molecular mechanisms that drive the process of body aging [36]. Replicative senescent MSCs dynamically change to senescence-related indicators, such as whole-map gene expression patterns and miRNA profiles. To identify biomarkers for senescent TMSCs, these changes should be considered therapeutic targets for MSC rejuvenation [37].

In this study, we established a senescent TMSC model in terms of morphological and biological characteristics *in vitro*. Senescent TMSCs were characterized by an enlarged and flattened cellular morphology by continuous subculture and increased SA-*β*-gal absorbance (Figures 1(a) and 1(b)). Along with the morphological changes, TMSC proliferation efficiency significantly decreased with replicative senescence and showed a prolonged G1 phase of the cell cycle and reduced S phase (Figures 1(c) and 1(d)). Moreover, our initial observations did not show changes in the hematopoietic and primitive markers between young and old TMSCs (Figure 2). The ability to detect changes in the expression of stem cell surface markers with senescence implies that more precise criteria are needed to identify senescent cells [17, 38].

We have recently reported the alteration of ITGA3, integrin *α*3 protein in the cell surface, with senescence through transcriptomic analysis from a previous study [11]. In the current study, we found that the percentage of G1 cells increased drastically during the senescence process, and this phenomenon has been studied as the “Hayflick phenomenon” in which cells lose their ability to proliferate [39]. Replicative senescence activates DNA damage responses and preferentially kills the stationary phase function of proteins and the majority of the proteins that fail to arrest growth in G0/G1 [14]. Thus, in this study, we aimed to determine the factors that delay the cell cycle and cell proliferation in TMSCs.

We confirmed the significantly changed genes of the two groups divided to determine the overall transcriptomic gene pool of TMSCs changed by replicative senescence (Figure 3, Table 1). Transcripts that are simultaneously involved in the cell cycle and cellular senescence were collected, and their classification was determined (Figure 3(a)). The expression of 18 transcripts, except HIPK2, was increased by replicative senescence. HIPK2 acts as a transcription factor that increases the DNA binding affinity and represses transcription [25, 30]; however, it was probably affected by the decrease in expression because the amount that could be used for DNA damage caused by the senescence of TMSCs was reduced.

Furthermore, according to the MF of the GO term with transcripts whose expression has changed (Figures 3(d)–3(f), Supplementary Table 2), DNA functions, including catalytic activity on DNA, DNA helicase activity, DNA replication origin binding, and DNA-dependent ATPase activity showed a significant difference in expression. Additionally, the cyclin-dependent protein kinase regulator GO term category was also found to be significant, and the greatest effect of senescence on MSC proliferation was investigated by cell cycle change due to DNA damage. The KEGG pathway also showed that cell cycle-related transcripts play an essential role in the MSC replicative senescence process (Figure 4 and Supplementary Figure 1).

A paradox in the cellular response to DNA damage is that a checkpoint is enforced by inhibiting CDK activity, whereas many studies report that CDK activity is needed in a DNA damage response for DNA replication, homologous recombination, and DNA repair [40]. Our study identified significant increases in the expression of CDK1 and CDK2 genes in senescent TMSCs, which are known as cell division cycle protein homologs that are highly conserved and are key players in cell cycle regulation [24, 41]. While the remaining CDK activity sustains important cellular functions in cell cycle, it also poses a risk for genome stability [42, 43]. If cells with damaged DNA progress, they need to be prevented from entering mitosis, which otherwise could result in chromosome missegregation and propagation of mutations [44]. Therefore, the activity of residual CDK present in senescent TMSCs can rather promote cellular senescence.

In the present study, the CDK family, which was not phosphorylated due to DNA damage from replicative senescence, could not bind to the cyclin partner, resulting in the increased percentage of cells in the G1 phase (Figure 1(d)). The increased G1 phase due to decreased CDK inhibited cell proliferation and eventually showed the “Hayflick phenomenon.” Quantitative gene expression and protein were also significantly increased in old TMSCs (Figures 4 and 5). Furthermore, cell proliferation efficiency was improved when CDK1 was inhibited/knocked down in old TMSCs (Figure 6). These findings suggest that a transcriptome-based approach could provide greater discriminating power for establishing new senescence biomarkers for MSCs studies and serve as a basis for regulating established CDK1-induced senescence mechanisms.

In contrast, TGFB2, which is a pleiotropic cytokine that regulates a myriad of cellular processes, was not changed in quantitative RT-PCR results. Mounting evidence shows that the effects of TGF-*β* on the proliferation and senescence of MSCs have varied in the literature [45, 46]. The TGF-*β* of senescence-promoting activity in several MSC types, including bone marrow MSCs and endometrial MSCs, has been previously described [47, 48]. In bone marrow MSCs, TGF-*β* reportedly increases the expression levels of p16lnk4a and 4-Hydroxynonenal subunits, SA-*β* gal activity, senescence markers, and the production of mitochondrial ROS [47]. However, other studies have suggested that TGF-*β* promotes the proliferation of MSCs or does not affect senescence [49]. These contradictory observations might be partly explained by the heterogeneity of MSCs, as human MSCs contain at least three types that possess differentiation capacities [5, 38].

Our transcriptomic analysis of senescent TMSCs revealed a much larger database, suggesting factors that can be selected for old MSCs. Future studies should investigate the detailed molecular mechanism, including changes in the intracellular localization of CDK1. It is necessary to check whether these results have the same effect in MSCs derived from other tissues. Additionally, further studies are needed to develop high-quality stem cell therapeutics through overall screening and mechanism research for various candidate substances capable of selecting senescent cells. These analyses have led us to propose that genes associated with the cell cycle and cellular senescence process are differentially altered with the aging of TMSCs, and some of these molecules could be used as potential indicators for identifying stem cells with replicative senescence.

## 5. Conclusions

In this study, we investigated transcriptomic changes in replicative senescent TMSCs compared to early passage TMSCs (young). We identified meaningful genes (CDK1 and CDK2) that consequently increased the proliferation of TMSCs. The CDK family could be used as an effective indicator or marker to improve quality control during isolation and expansion of MSCs, thereby increasing the therapeutic efficacy of MSC-based regenerative therapy. However, further investigations are required to fully evaluate the effectiveness and detailed molecular mechanisms, including changes in the intracellular localization of the CDK family. Additionally, other molecules classified into different GO term categories and related regulatory mechanisms remain to be identified.

## Figures and Tables

**Table 1:** List of cell cycle related-heatmap component genes.

Description	Old/young TMSCs fc.	Old/young TMSCs fc.sd
Transforming growth factor beta 2	2.031802	0.372581093
E2F transcription factor 1	2.38874	0.294603229
Retinoblastoma-like 1	2.679363	0.27475041
Major histocompatibility complex, class I, C	2.046949	0.406833552
ATR serine/threonine kinase	2.076682	0.530629568
v-myb avian myeloblastosis viral oncogene homolog-like 2	2.718828	0.395853769
MRE11 homolog A, double-strand break repair nuclease	2.672407	0.531469337
Cyclin A2	4.441802	0.785618372
Forkhead box M1	3.543905	0.72503185
Cyclin B2	6.951431	0.590018707
Calcium channel, voltage-dependent, L type, alpha 1D subunit	2.125666	0.119489555
Cyclin E2	5.824786	0.304741692
Cyclin B1	4.1434	0.269147843
Cyclin-dependent kinase 1	8.146046	0.084295292
v-ets avian erythroblastosis virus E26 oncogene homolog 1	2.277925	0.181972532
Cyclin-dependent kinase 2	3.047835	0.284278114
Lin-9 DREAM MuvB core complex component	2.36989	0.20922594
Cell division cycle 25A	2.483991	0.696660816
Homeodomain-interacting protein kinase 2	-2.595729	0.243196895

ATR: ataxia telangiectasia and Rad3 related; MRE: meiotic recombination 11.

## Data Availability

The data that support the findings of this study are available upon request from the corresponding author. The data are not publicly available due to privacy concerns or ethical reasons.

## Supplementary Materials

Supplementary Materials Supplementary Figure 1: KEGG pathway classification map of the experimental group. KEGG enrichment map analysis evaluated changes in the expression of genes related to the cellular senescence. Supplementary Table 1: general characteristics of donors. Supplementary Table 2: genes are included in the three subcategories of the KEGG pathway map. All genes in the three subcategories that showed the most significant differences (*p* < 0.001) in the KEGG pathway in Figure 5 are listed.
