# Supplementary material for: Cyclin-Dependent Kinase 1 Inhibition Potentiates the Proliferation of Tonsil-Derived Mesenchymal Stem Cells by Delaying Cellular Senescence
Source: Stem Cells Int. 2022 Jul 21;2022:4302992. doi: 10.1155/2022/4302992 (PMC9337930; doi:10.1155/2022/4302992)
Supplement: Supplementary Materials — Supplementary Figure 1: KEGG pathway classification map of the experimental group. KEGG enrichment map analysis evaluated changes in the expression of genes related to the cellular senescence. Supplementary Table 1: general characteristics of donors. Supplementary Table 2: genes are included in the three subcategories of the KEGG pathway map. All genes in the three subcategories that showed the most significant differences (p < 0.001) in the KEGG pathway in Figure 5 are listed. [file 4302992.f1.docx]

**Supplementary Figure and Table**


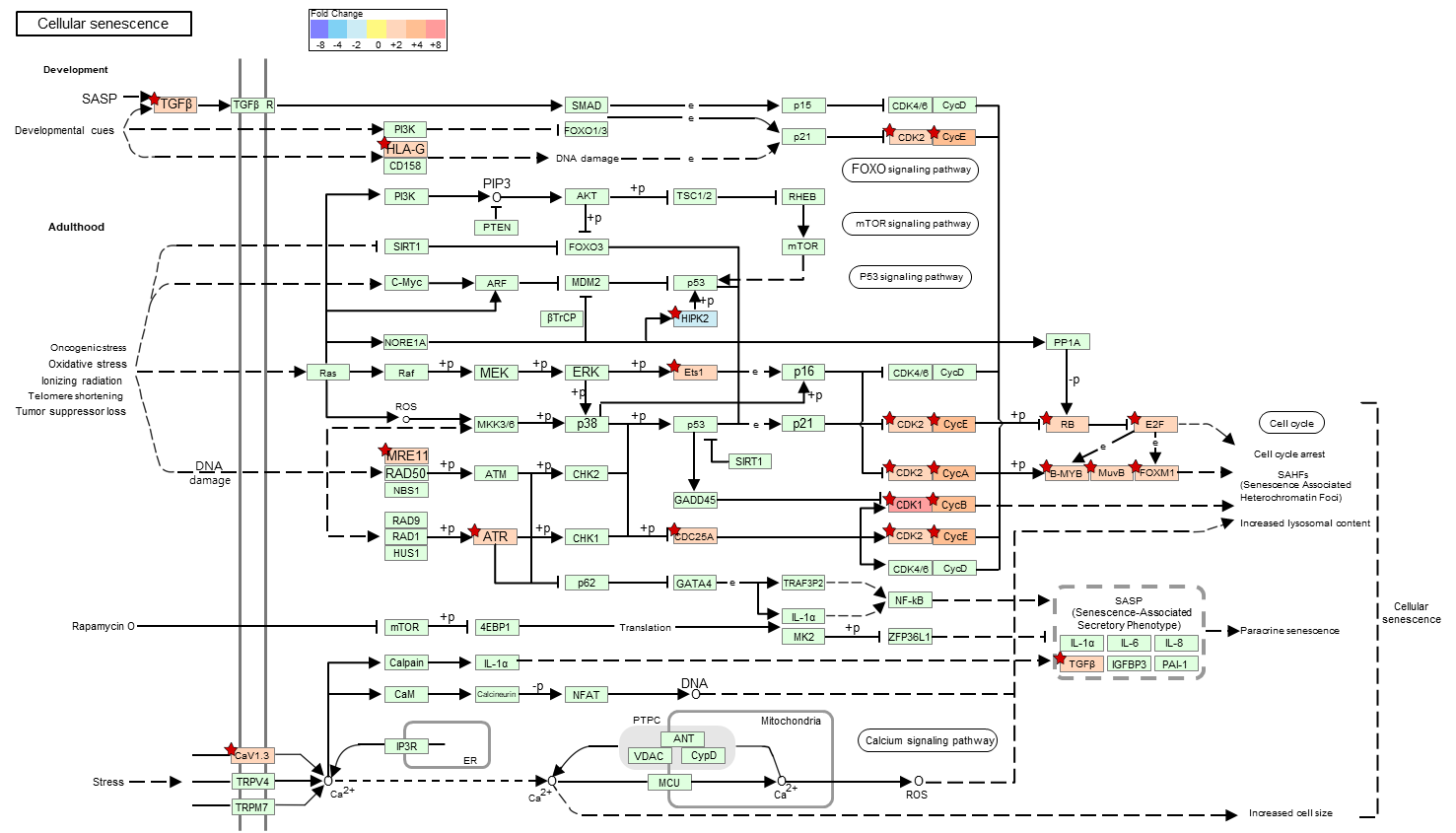


Supplementary Figure 1. KEGG pathway classification map of the experimental group. KEGG enrichment map analysis evaluated changes in the expression of genes related to the cellular senescence.

| **No.** | **Sex** | **Age** | **Height (cm)** | **Weight (kg)** | **Tonsil weight (g)** |
| --- | --- | --- | --- | --- | --- |
| **1** | Male | 4 | 116.7 | 21.5 | 1.46 |
| **2** | Male | 6 | 116.9 | 22.9 | 1.34 |
| **3** | Female | 5 | 109.1 | 23.2 | 1.29 |
| **4** | Female | 7 | 124.7 | 23.4 | 1.68 |

Supplementary Table 1. General characteristics of donors

Supplementary Table 2. Genes are included in the three sub-categories of the KEGG pathway map

| **Map Name** | **No. of Significant gene** | **Gene Symbol** | **Up-regulated genes  (Old /Young TMSCs fc. (+))** | **Down-regulated genes (Old/Young TMSCs fc. (-))** | ***P-*value** |
| --- | --- | --- | --- | --- | --- |
| Cellular senescence | 19 | *TGFB2, LIN9, CACNA1D, CDC25A, ATR, CCNA2, CCNB1, HLA-C, HIPK2, CCNE2, CDK1, MRE11A, ETS1, CDK2, FOXM1, CCNB2, MYBL2, E2F1, RBL1* | *TGFB2, LIN9, CACNA1D, CDC25A, ATR, CCNA2, CCNB1, HLA-C, CCNE2, CDK1, MRE11A, ETS1, CDK2, FOXM1, CCNB2, MYBL2, E2F1, RBL1* | *HIPK2* | 2.93E-09 |
| p53 signaling pathway | 11 | *RRM2, STEAP3, CASP8, ATR, CCNB1, CCNE2, CDK1, SESN3, CDK2, CCNB2, GTSE1* | *RRM2, ATR, CCNB1, CCNE2, CDK1, CDK2, CCNB2, GTSE1* | *STEAP3, CASP8, SESN3* | 2.48E-06 |
| Cell cycle | 35 | *CDC20, CDKN2C, CDC7, TGFB2, MAD2L2, ORC1, BUB1, MCM6, CDC25A, ATR, MAD2L2, CCNA2, CCNB1, PTTG1, CDC25C, TTK, MCM3, DBF4, MCM7, MCM4, CCN2, RAD21, CDK1, ESPL1, CDK2, CCNB2, BUB1B, PLK1, ORC6, CDC6, PCNA, E2F1, RBL1, CDC45, MCM5* | *CDC20, CDKN2C, CDC7, TGFB2, MAD2L2, ORC1, BUB1, MCM6, CDC25A, ATR, MAD2L2, CCNA2, CCNB1, PTTG1, CDC25C, TTK, MCM3, DBF4, MCM7, MCM4, CCN2, RAD21, CDK1, ESPL1, CDK2, CCNB2, BUB1B, PLK1, ORC6, CDC6, PCNA, E2F1, RBL1, CDC45, MCM5* | *-* | 7.39E-27 |
